# The proteasome inhibitor MG-132 sensitizes PC-3 prostate cancer cells to ionizing radiation by a DNA-PK-independent mechanism

**DOI:** 10.1186/1471-2407-5-76

**Published:** 2005-07-07

**Authors:** Frank Pajonk, Arndt van Ophoven, Christian Weissenberger, William H McBride

**Affiliations:** 1 Department of Radiation Oncology, David Geffen School of Medicine at UCLA, 10833 Le Conte Avenue, Los Angeles, CA90095-1714, USA; 2 Department of Urology, University Hospital Münster, Albert-Schweitzer-Straße 33, D-48149 Münster Germany; 3 Department of Radiation Oncology, University Hospital Freiburg, Robert-Koch-Straße 3, D-79106 Freiburg, Germany

## Abstract

**Background:**

By modulating the expression levels of specific signal transduction molecules, the 26S proteasome plays a central role in determining cell cycle progression or arrest and cell survival or death in response to stress stimuli, including ionizing radiation. Inhibition of proteasome function by specific drugs results in cell cycle arrest, apoptosis and radiosensitization of many cancer cell lines. This study investigates whether there is also a concomitant increase in cellular radiosensitivity if proteasome inhibition occurs only transiently before radiation. Further, since proteasome inhibition has been shown to activate caspase-3, which is involved in apoptosis, and caspase-3 can cleave DNA-PKcs, which is involved in DNA-double strand repair, the hypothesis was tested that caspase-3 activation was essential for both apoptosis and radiosensitization following proteasome inhibition.

**Methods:**

Prostate carcinoma PC-3 cells were treated with the reversible proteasome inhibitor MG-132. Cell cycle distribution, apoptosis, caspase-3 activity, DNA-PKcs protein levels and DNA-PK activity were monitored. Radiosensitivity was assessed using a clonogenic assay.

**Results:**

Inhibition of proteasome function caused cell cycle arrest and apoptosis but this did not involve early activation of caspase-3. Short-time inhibition of proteasome function also caused radiosensitization but this did not involve a decrease in DNA-PKcs protein levels or DNA-PK activity.

**Conclusion:**

We conclude that caspase-dependent cleavage of DNA-PKcs during apoptosis does not contribute to the radiosensitizing effects of MG-132.

## Background

One of the most challenging ongoing efforts in radiotherapy is the search for agents that target tumor-specific characteristics to cause radiosensitization without increasing normal tissue complications. The malignant phenotype is normally associated with acquisition of mutations in genes encoding signal transduction molecules that control cell proliferation and/or cell death. Numerous experimental studies have shown, that the expression of mutated oncogenes and tumor suppressor genes in normal cells alters their intrinsic cellular radiosensitivity and clinical studies have, in some cases, indicated that such mutations influence disease free survival after chemotherapy and radiation therapy [1-4]. These observations suggest that the pathways leading to cell cycle arrest and cell death following exposure to ionizing irradiation are linked to DNA damage and repair. Our knowledge of the molecular circuitry that is involved is still however far from complete.

One of the processes that controls expression of short-lived cell cycle and cell death regulators and transcription factors, such as cyclin A, B and E, p21 and p27, p53, cJun, cFos, and nuclear factor κB (NF-κB), is the proteolytic degradation through the 26S proteasome [5-7]. Indeed, the initial response to many stress signals such as radiation-induced DNA damage [8], hypoxia/reperfusion [9], and exposure to heat [10,11] or cytokines [12] appears to involve rapid alterations in 26S proteasome activity resulting in cell cycle arrest and/or apoptosis.

The 26S proteasome is a 2 MDa proteolytic complex that has 3 different cleavage activities [13]. Its function is highly regulated [14-18]. Highly specific and potent inhibitors of the 26S proteasome like the reversible inhibitor MG-132 or lactacystein, which acts irreversibly, have been shown to induce apoptosis in many tumor cell lines [19-27] and in SV-40-transformed but not normal human fibroblasts [27]. Apoptosis is regulated by the ubiquitin/proteasome system at various levels and inhibition of proteasome function may induce [21] or prevent apoptosis [28]. A possible association between apoptosis and radiosensitization exist through caspase activation and subsequent proteolytic destruction of DNA repair enzymes. Caspase activation has been reported to follow proteasome inhibition [24,29,30] and is known to degrade the catalytic subunit of DNA-dependent protein kinase (DNA-PKcs), a key DNA repair enzyme of non-homologous end-joining (NHEJ) of DNA double strand breaks [31,32].

Still, the exact mechanisms of how proteasome inhibition causes apoptosis are not fully understood but the fact, that malignant cells are much more sensitive to the death-promoting aspects of proteasome inhibition than normal cells [33] makes the ubiquitin/26S proteasome system a target for cancer therapy. With Velcade (formerly known as bortezomib, PS-341) a first proteasome inhibitor has become available for clinical use [34]. The excellent results achieved in patients with multiple myeloma suggest the use of Velcade in solid cancers, especially in combination with classical chemotherapies or radiation therapy. So far, several Phase I/II studies have used Velcade as mono-therapy in advanced chemotherapy refractory solid cancers [35-40] but knowledge of in-vivo effects of combined modality approaches using Velcade is limited. It is known that proteasome inhibition can sensitize cells to chemotherapy [41] and ionizing radiation [26,42] and therefore combination studies will be launched in the near future.

However, a recent *in-vitro *report demonstrated that Velcade treatment of A549 lung cancer might enhance or attenuate cisplatin toxicity, depending on the sequence of application of both drugs [28]. These findings underline the need for a better understanding of the mechanisms of action of these new compounds as they may interfere with standard therapies.

In this study, we investigated a possible link between induction of the apoptotic death program and radiosensitization following proteasome inhibition.

## Methods

### Cell culture

Cultures of PC-3 prostate carcinoma cells American Type Culture Collection (ATCC, Manassas) cells were grown in 75 cm^2 ^flasks (Falcon) at 37°C in a humidified atmosphere at 5 % CO_2_. PC-3 cells were cultured in DMEM medium (Cell Concepts, Umkirch Germany) supplemented with 10 % heat-inactivated fetal calf serum (FCS, Sigma, St. Louis, MO) and 1 % penicillin/streptomycin (Sigma).

### MG-132 treatment

MG-132 (Calbiochem, San Diego, CA) was dissolved in DMSO (10 mM) and small aliquots (30 μl) were stored at -20°C. Velcade (Janssen-Cilag, Neuss, Germany) was solubilized in water at a concentration of 1 mg/ml and stored in aliquots of 50 μl at -80°C. Three hours before irradiation growth medium was replaced by medium containing MG-132 (50 μM, 0.5% DMSO) or Velcade (100 nM in water). Control cells were subjected to DMSO treatment alone (0.5 %). In clonogenic assays, cells were incubated at 37°C for 3 hours, washed with DMEM, trypsinized, counted, diluted and irradiated at room temperature.

### Irradiation

Exponentially growing PC-3 cells were trypsinized, counted, and diluted. The cell suspensions were immediately irradiated at room temperature using a ^137^Cs-laboratory irradiator (JLShephard, Mark I) at a dose rate of 5.80 Gy/min and a Gammacell 40 ^137^Cs-laboratory irradiator at a dose rate of 0.83 Gy/min respectively.

### Clonogenic survival

Colony-forming assays with PC-3 cells were performed immediately after irradiation by plating an appropriate number of cells into culture dishes, in triplicate. Before plating, the viability of the cells was assessed during counting by a dye exclusion test with tryphan blue. After 14 days, colonies were fixed with methanol, stained with crystal violet, and were counted if they consisted of more than 50 cells. The fraction of cells surviving irradiation was normalized to the surviving fraction of the corresponding control and survival values and curves were fitted to the data using a linear-quadratic model.

### Flow cytometry

For analysis of cell cycle distribution, 1 × 10^5 ^cells were trypsinized, washed in PBS and fixed with ice-cold ethanol (70 %). After RNAse treatment (1 mg/ml), cells were permeabilized with Triton X-100 and stained with propidium iodide (0.1 mg/ml). To determine the cell cycle distribution, DNA content was measured using a flow cytometer (FACScan, Becton Dickinson). TUNEL-Assay was carried out as described previously [43].

### DNA-PK activity assay

In order to assess DNA-PK activity, DNA-PK-dependent phosphorylation of a biotinylated p53-derived peptide (Glu-Pro-Pro-Leu-Ser-Gln-Glu-Ala-Phe-Ala-Asp-Leu-Trp-Lys-Lys. Promega, Madison, WI; [44]) was measured in the presence of [^32^P-γ]-ATP. Drug-treated or control cells were washed in low salt buffer (10 mM HEPES, 25 mM KCl, 10 mM NaCl, 1.1 mM MgCl_2_, 0.1 mM ethylenediaminetetraacetic acid [EDTA], 0.5 mM phenylmethanesulfonyl fluoride [PMSF], pH 7.2), pelleted and lysed by one freezing/thawing cycle as described in [45]. After centrifugation at 10,000 × g for 5 minutes at 4°C, the supernatant was collected and used as cell extract. Protein content was determined using the Micro-BCA protocol (Pierce) with bovine serum albumin (Sigma) as standard. 10 μg protein were incubated for 30 minutes at 30°C in DNA-PK reaction buffer (50 mM HEPES (KOH, pH 7.5), 100 mM KCl, 10 mM MgCl_2_, 0.2 mM Ethylene glycol-bis(2-aminoethylether)-*N,N,N',N'*-tetraacetic acid [EGTA], 0.1 mM EDTA, 1 mM DTT), 0.025 mM ATP, 0.5 μCi [^32^P-γ]-ATP, bovine serum albumin [BSA] 0.1 mg/ml, and with human p53 oligopeptide as substrate in the presence or absence of activated calf thymus DNA to measure DNA-DSB-dependent phosphorylation of p53. The final volume was 25 μl. The reaction was stopped by addition of 25 μl 30 % acetic acid. 10 μl was spotted on Whatman P81 membranes in duplicates and washed four times with 15 % acetic acid.

Membranes were placed on a phosphor imager screen for 2 hours. The screen was read on a phosphor imager (Storm 860, Molecular Dynamics) and the activity measured using the ImageQuant software package (Molecular Dynamics). Activity in the absence of activated DNA was assumed to be unspecific and thus subtracted from corresponding measurements in presence of activated DNA.

### Caspase-3 activity assay

For assessment of caspase-3 activity, cells were plated into culture dishes 24 hours before drug treatment. After drug treatment, cells were dislodged mechanically and washed twice in PBS. Caspase-3 activity was assessed as described by Enari et al. [46] with minor modifications: After five cycles of freezing and thawing in extraction buffer (50 mM PIPES-NaOH, pH 7.0, 50 mM KCl, 5 mM EGTA, 2 mM MgCl_2_, 1 mM dithiothreitol [DTT], 20 μM cytochalasin B, 1 mM phenylmethylsulfonyl fluoride [PMSF], 1 μg/ml leupeptin, 1 μg/ml pepstatin A, 50 μg/ml antipain, 10 μg/ml chymopain) lysates were centrifuged at 10,000 × g for 12 minutes (4°C). The supernatant was immediately frozen in liquid nitrogen and stored at -80°C. Protein concentrations were determined using the Micro-BCA protocol (Pierce) with bovine serum albumin (Sigma) as standard. 36 μg protein were diluted in ICE standard buffer (100 mM HEPES-KOH, pH 7.5, 10 % sucrose, 0.1 % CHAPS, 10 mM DTT, 0.1 mg/ml ovalbumin) containing the fluorogenic caspase-3 substrate DEVD-7-amido-4-methylcoumarin (DEVD-AMC, 1 μM) and incubated for 30 minutes at 30°C. Fluorescence was measured using a fluorescence plate reader (Tecon, excitation 380 nm, emission 460 nm).

### Immunoblotting

Cells were washed with PBS and lysed in TOTEX buffer (20 mM HEPES [pH 7.9], 0.35 mM NaCl, 20 % glycerol, 1 % Nonidet P-40, 0.5 mM EDTA, 0.1 mM EGTA, 0.5 mM DTT, 50 μM PMSF and 90 trypsin inhibitor units [TIU's]/ml aprotinin) for 30 minutes on ice. The lysate was centrifuged at 12,000 × *g *for 5 minutes and the supernatant transferred to fresh tubes. Protein concentrations were determined using the Micro-BCA protocol (Pierce, Rockford, IL) with bovine serum albumin as standard. 100 μg or 50 μg of protein were subjected to SDS gel electrophoresis (0.1 % Sodium dodecyl sulfate [SDS]/6 % polyacrylamide) and blotted to PVDF membranes. Equity of protein loading was confirmed by Ponceau staining of the PVDF membranes. After blocking with 5 % skim milk in PBS, membranes were incubated with a mouse polyclonal antibody against human DNA-PKcs (Santa Cruz Biotechnologies). A secondary horseradish-peroxidase-conjugated antibody and the ECL Plus System (Amersham) were used for visualization. Fluorescence was measured using a Typhoon 9410 Phosphorimager (Blue fluorescence, 600 V, Amersham).

## Results

### MG-132 induces apoptosis and sensitizes PC-3 cells to ionizing radiation

**Figure 1:**
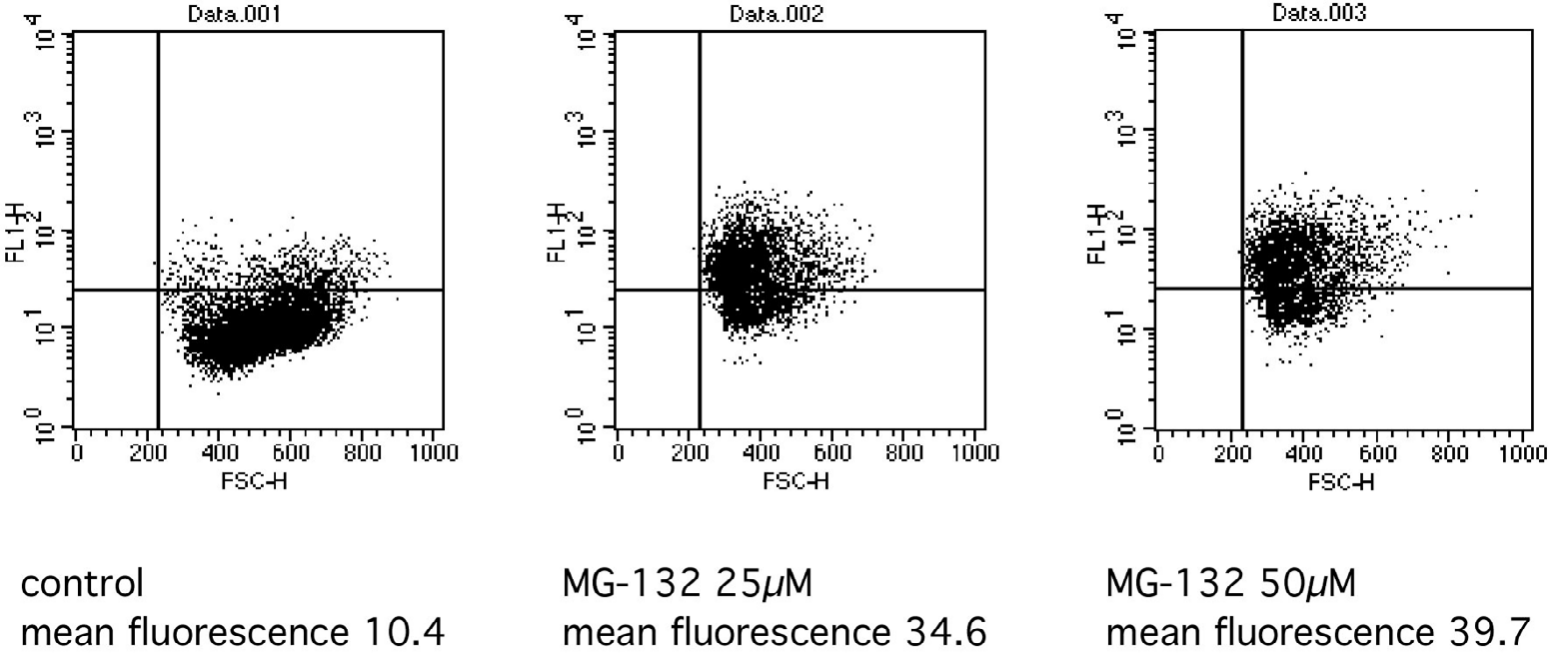
*MG-132 induces apoptosis in PC-3 cells*. Representative (n = 3) FACS analysis of PC-3 prostate cancer cells incubated with 0, 25 and 50 μM concentrations of MG-132 for 24 h. TUNEL-staining indicated an increase of apoptotic cells from 7.6 % in control cells to 66.5 % and 72.8 % in MG-132 treated cells.

To investigate if transient proteasome inhibition by proteasome inhibition affects PC-3 cells induction of apoptosis and long-term clonogenic survival was examined following MG-132 treatment. Cells were exposed to 50 μM of MG-132 for 3 hours, washed twice, trypsinized and plated into tissue culture dishes in triplicates. Clonogenicity, as assessed by colony counts after 2 weeks incubation, was decreased by MG-132 treatment of PC-3 cells from 65 ± 5.1 % in dimethyl sulfoxide (DMSO)-treated versus 35 ± 2.1 in MG-132-treated cells. Decreased clonogenicity was due to apoptosis, as assessed morphologically. 3 hours exposure of PC-3 cells to MG-132 at 25 μM and 50 μM concentrations increased the TUNEL-positive (terminal deoxynucleotidyl transferase-mediated nick end labeling) population after 24 hours of incubation from initially 8 % to 67 % and 73 % respectively (Fig. 1). Short-term proteasome inhibition for 3 hours was therefore effective in causing apoptosis in a proportion of cells, but a considerable number remained clonogenically viable.

**Figure 2:**
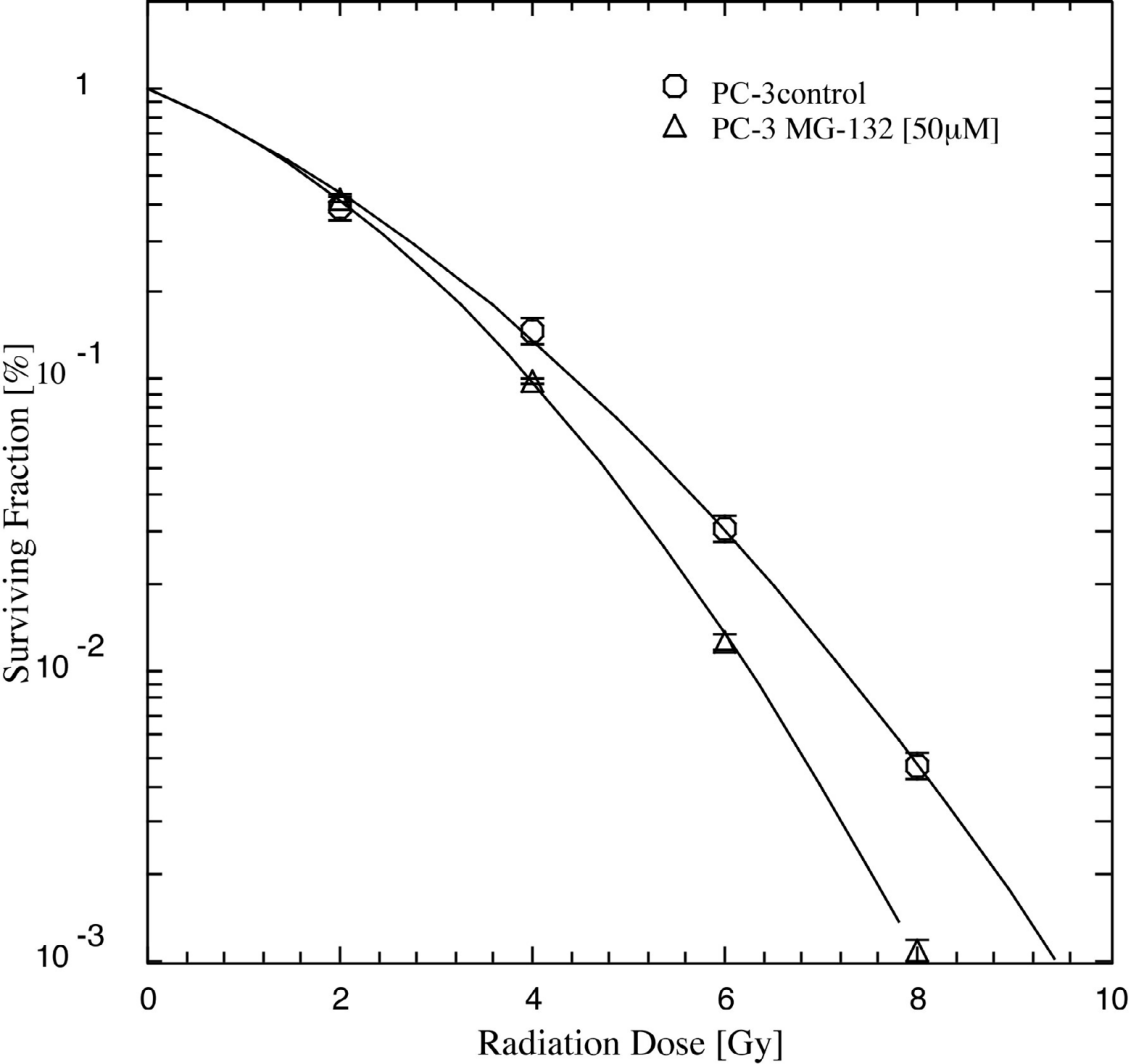
*MG-132 sensitizes human cancer cell lines to ionizing radiation*. PC-3 prostate cancer cells were incubated with MG-132 (50 μM) for 3 hours, washed twice, irradiated and plated into culture dishes. After 14 days the colonies were fixed with ethanol, stained with crystal violet, and counted. MG-132 treatment sensitized PC-3 cells to ionizing radiation. Radiobiological parameters obtained from a linear-quadratic fit (LQ-fit): PC-3 control α = 0.3, β = 0.043, α/β = 7.6; PC-3 MG-132 α = 0.3, β = 0.07, α/β = 4.3. Data shows means (± standard deviation) from 3 experiments (each plated in triplicates).

Clinical treatment with proteasome inhibitors might also be expected to spare a proportion of clonogenic tumor cells and it would be an advantage if these were sensitized to some other form of therapy. To test whether they were sensitized to radiation, PC-3 cells were exposed to short-term treatment with MG-132. After 3 hours, cells were washed, irradiated and plated in a clonogenic assay, as described previously [43]. Inhibition of proteasome function by MG-132 sensitized the surviving clonogenic PC-3 cells to the effects of ionizing radiation as shown by the left-shift of the survival curve (Fig. 2).

### Effect of 26S proteasome inhibition on radiation-induced cell cycle arrest and apoptosis

Having validated PC-3 cells as a model for radiosensitization by proteasome inhibitors, we first excluded that the observed radiosensitizing effect was a result of changes in cell cycle distribution. Inhibition of 26S proteasome function is known to block cellular transition from G1- to S-phase and from late S- to G2/M-phase, as well as S phase transition [47]. This is considered to be due to alterations in expression of molecules such as p53, p21^WAF/CIP1^, pRB, p27, and cyclins A, B, and E. Similar effects can be seen following exposure of cells to ionizing radiation, which can cause arrest at the G1 and G2/M checkpoints, as well as S-phase delay, and apoptosis in certain cancer cell lines [48]. In cells with mutated p53, like PC-3 prostate cancer cells, the radiation-induced G1 checkpoint delay and the pro-apoptotic response are often abrogated, but the cells will arrest in G2/M (reviewed in [49]).

**Figure 3:**
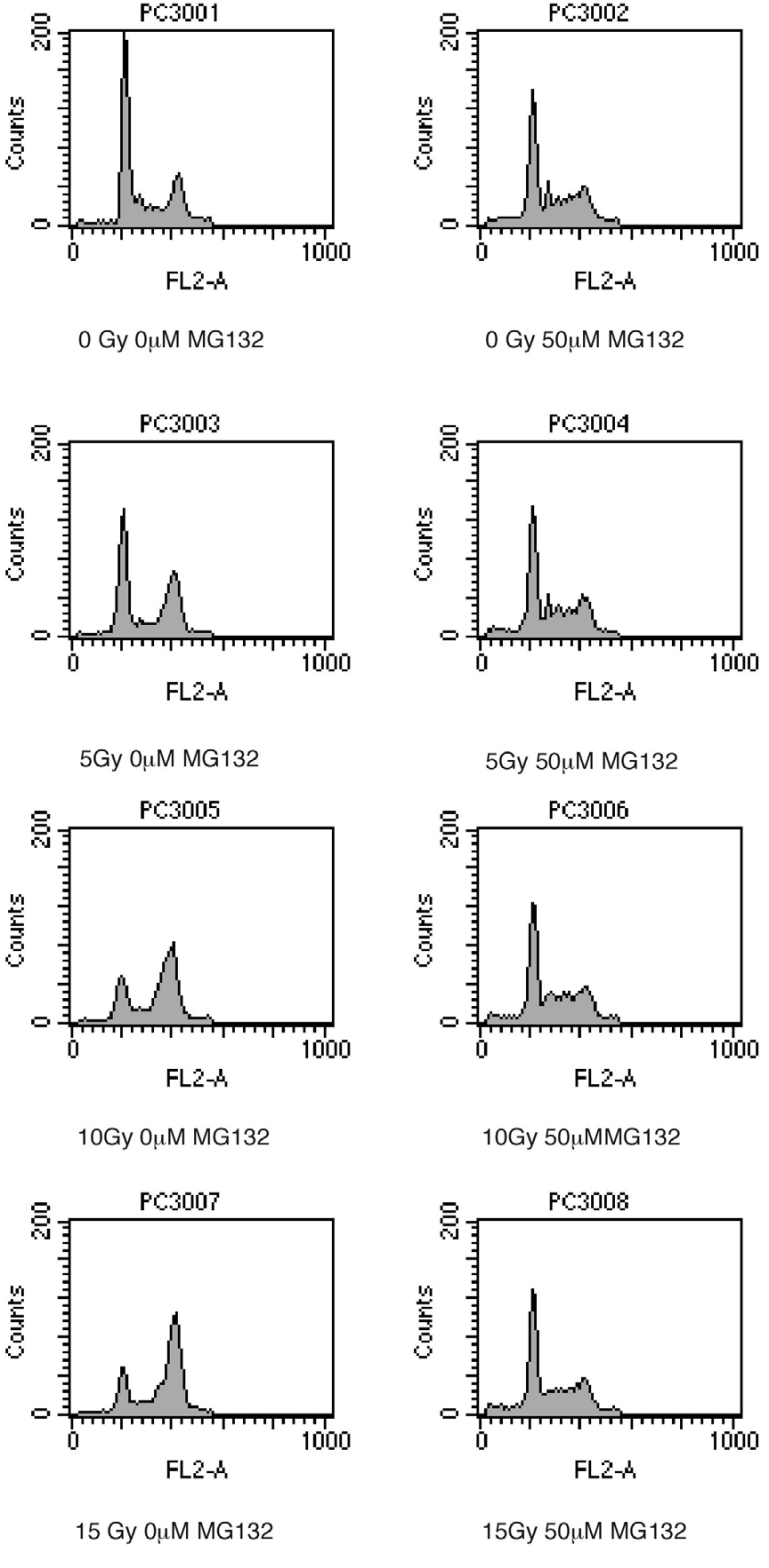
*The effect of proteasome inhibition by MG-132 on cell cycle progression and radiation-induced cell cycle arrest and apoptosis*. Cell cycle distribution of PC-3 prostate cancer cells assessed by flow cytometry after staining with propidium iodide. Cells were pre-incubated with MG-132 (50 μM) for 3 hours, irradiated, and incubated for additional 24 hours in the presence of MG-132. MG-132 prevented cell cycle progression and, consequently, dose-dependent radiation-induced G2/M-arrest. Additionally, MG-132 treatment increased the number of cells in late S-phase.

The effect of the combined treatment of proteasome inhibitors and ionizing irradiation on cell cycle arrest was tested using PC-3 cells treated continuously with MG-132 (50 μM) from 3 hours prior up to 24 hours after irradiation. This treatment blocks proteasome activity almost completely [50]. Flow cytometric analyses indicated that radiation induced G2/M but not G1 arrest, as would be expected in the p53-null PC-3 cells (Fig. 3). MG-132 treatment caused p53-independent cell cycle arrest in all phases and, as a result, the combination of both treatments did not lead to a radiation-induced G2/M arrest (Fig. 3).

### The effect of MG-132 treatment on caspase-3 and DNA-PK activities

Since short-term proteasome inhibition caused both apoptosis and radiosensitization in PC-3 cells, this model can be used to investigate the relationship between these phenomena and to explore the pathways that might interconnect them. We considered the hypothesis that both involve caspase-3 activation. Proteasome inhibition has been reported to induce apoptosis that is mediated by caspase activation [20]. Also, the catalytic subunit of the DNA-PK complex, DNA-PKcs, which is required for NHEJ repair pathway of DNA double strand breaks, is a known substrate of caspase-3 [51]. Its destruction could result in radiosensitization.

**Figure 4:**
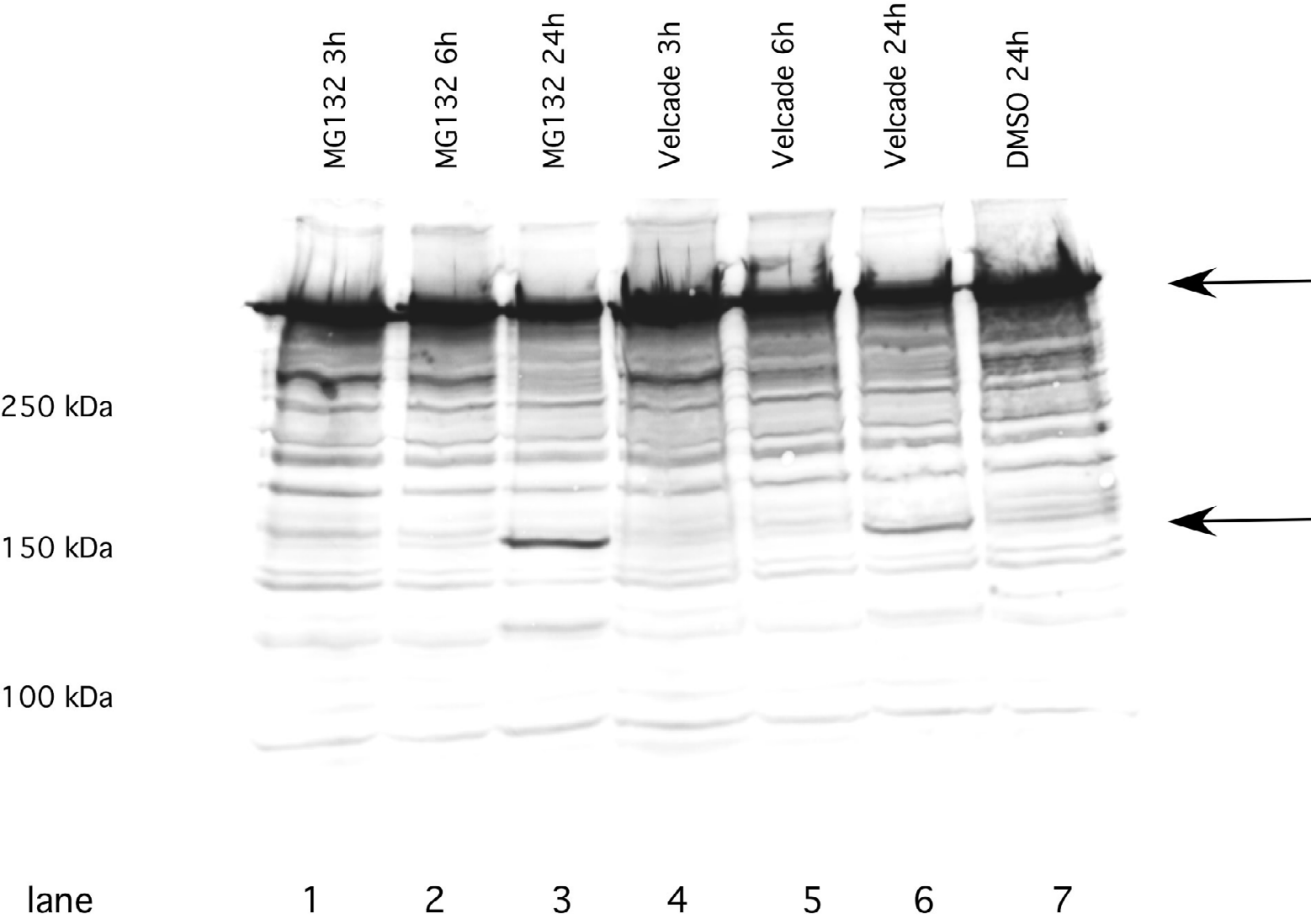
*Degradation of DNA-PKcs by caspase-3 is a late event in apoptosis induced by proteasome inhibition in PC-3 cells*. Representative (n = 3) western blot of lysates from PC-3 prostate cancer cells after 3, 6 and 24 hours incubation with MG-132 (50 μM) or Velcade (100 nM) using a specific antibody against human DNA-PKcs. The antibody recognizes the intact 460 kDa protein as well as the specific 160 kDa fragment observed after caspase-3 dependent cleavage of DNA-PKcs. MG-132 caused no significant change of the DNA-PKcs protein levels over a period of 6 hours. Detection of a caspase-3-cleavage specific 160 kDa fragment coincided with apoptosis at 24 hours, but did not occur during the first 6 hours.

MG-132 (50 μM) was maintained in the growth medium of PC-3 cells for various time periods. DNA-PKcs protein levels were monitored by western blotting (Fig. 4). Expression of the intact 460 kDa (upper arrow) protein did not change after 3 (lane 1) and 6 hours (lane 2) but slightly decreased after 24 hours of incubation (lane 3) compared to DMSO-treated control cells (lane 7). At this time-point the specific 160 kDa caspase-3-dependent degradation product of DNA-PKcs appeared (lane 3, lower arrow) which coincided with occurrence of morphological signs of apoptosis. Comparable results were obtained using Velcade (100 nM) another specific proteasome inhibitor (lane 4,5 and 6, Fig. 4).

**Figure 5:**
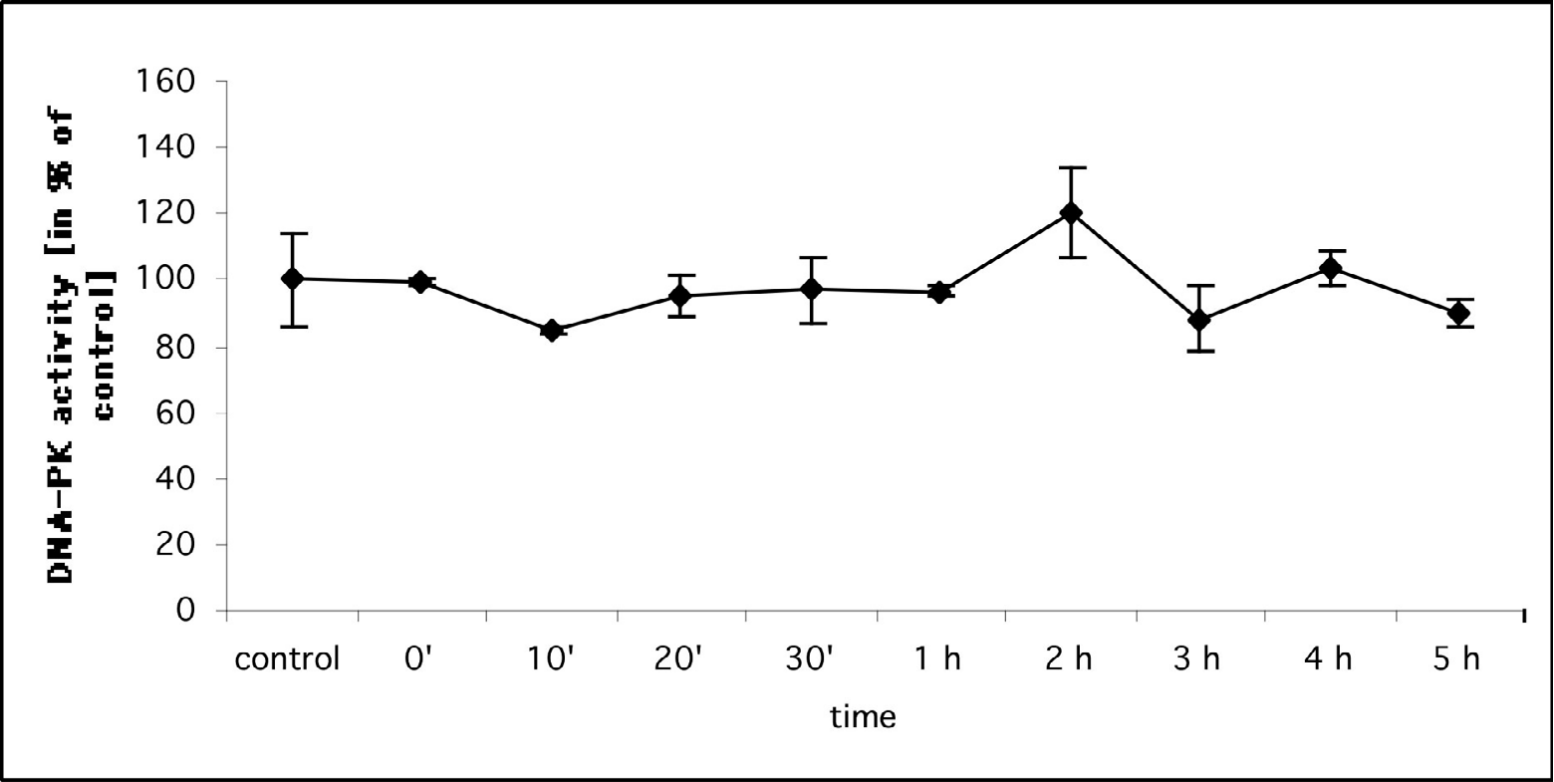
*MG-132 does not decrease DNA-PK activity*. PC-3 cells were incubated for 3 hours with MG-132 (50 μM). Cells were washed and incubated at 37°C. At indicated times, cells were lysed and DNA-PK activity was measured by phosphorylation of a human p53 derived oligopeptide in the presence of DNA DSB's. Proteasome inhibition did not cause any significant decrease of DNA-PK activity (n = 2, data expressed as mean ± standard error mean).

**Figure 6:**
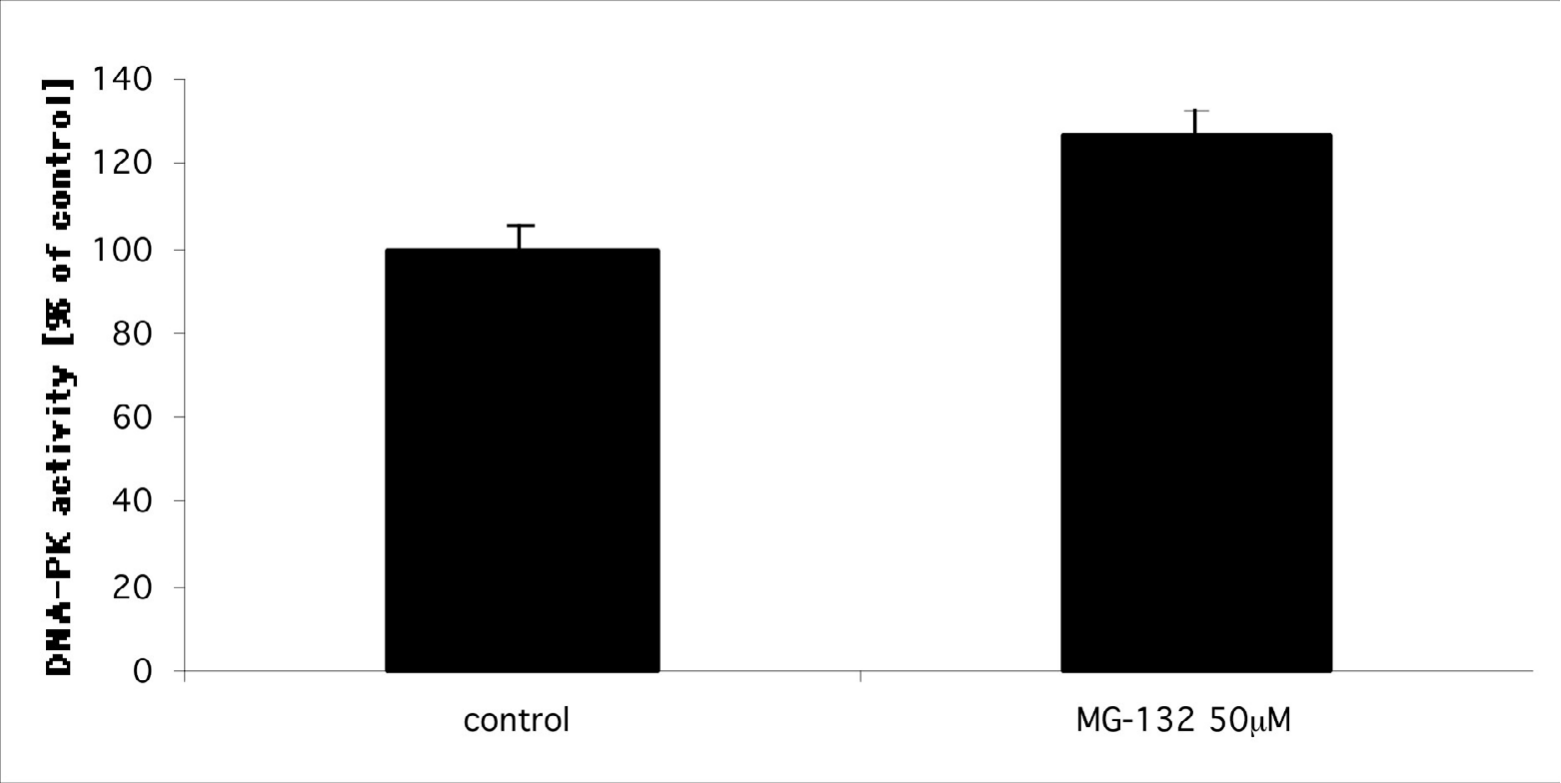
*MG-132 does not interfere with DNA-PK directly*. Lysates of untreated PC-3 cells were incubated with MG-132. There was no decrease in DNA-PK activity, excluding any direct interaction with the drug (n = 3, data expressed as mean ± standard error mean).

**Figure 7:**
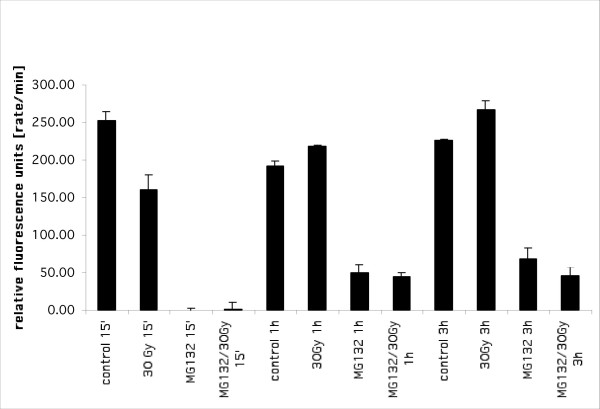
*MG-132 treatment reduces caspase-3-like activity in PC-3 cells*. PC-3 cells were pre-incubated with MG-132 (50 μM) for 3 hours, washed, irradiated, and incubated at 37°C. 15 minutes, 1, and 3 hours later, cells were lysed and total cellular protein was assayed for caspase-3-like activity, measuring the release of AMC from the fluorogenic caspase-3 substrate DEVD-AMC. Irradiation with 30 Gy initially decreased constitutive caspase-3 activity in PC-3 cells at 15 minutes followed by a slight increase at 1 and 3 hours after irradiation. In contrast, proteasome inhibition with MG-132 completely inhibited caspase-3 activity at 15 minutes. Activity recovered slowly reaching about 30% of baseline levels after 3 hours. This was not altered by combination of MG-132 treatment with ionizing radiation (n = 3, data expressed as mean ± standard error mean).

Because the level of DNA-PKcs might not reflect the total kinase activity of the enzyme complex, functional activity was assessed by phosphorylation of a p53 protein fragment in the presence of DNA double strand breaks. This was unchanged over a period of 5 hours after MG-132 treatment, which is the period over which most DNA repair would be expected to take place (Fig 5). In order to exclude a possible trivial explanation that MG-132 might directly interfere with DNA-PK activity, lysates of PC-3 cells were incubated with MG-132 and studied for p53 phosphorylation. The drug did not affect DNA-PK activity directly (Fig. 6). Since caspase-3 activity has been reported to be increased by proteasome inhibition in the MOe7 cell line [20], caspase-3 activity was measured using a fluorogenic substrate in PC-3 cells at various time points after short-term (3 hours) treatment of PC-3 cells with MG-132. We found a substantial drop in caspase-3-like activity as early as 15' minutes after the end of MG-132 incubation if compared to untreated control cells. Caspase-3-like cleavage activity slowly recovered about 3 hours after the end of MG-132 incubation (Fig. 7), but was never increased above baseline. Radiation (30 Gy) with or without MG-132 pre-treatment also failed to activate caspase-3-like activity during the first 3 hours.

## Discussion

Proteasome inhibitors like MG-132 sensitize cancer cells to ionizing radiation. In this study, we investigated interference of proteasome inhibition with NHEJ by caspase-3-dependent cleavage of DNA-PKcs as a possible underlying mechanism.

Treatment with MG-132 caused cell cycle arrest in un-irradiated and irradiated cells, induced apoptosis, decreased clonogenicity, and sensitized the surviving cells to ionizing radiation. This was in accordance with previous reports [19-21,47,52-55].

Drug-induced changes in cell cycle redistribution were most unlikely responsible for this effect because even after 24 hours MG-132 treatment mainly increased the number of cells in late S-phase, which has been shown to coincide with greatest radioresistance [56].

The relationship between pro-apoptotic pathways and radiosensitization is controversial, as is the importance of apoptosis in cell death following exposure to clinically relevant doses of ionizing radiation. In general, the majority of cells in solid carcinomas do not enter the apoptotic death pathway [57]. Instead, they undergo several cell divisions until they die or finally survive. Thus, the fate of cells in solid carcinomas after irradiation may be determined more by their ability to repair DNA damage caused by ionizing radiation than by initiation of apoptosis [58]. However, common molecular pathways may link these two phenomena. So far, any link between survival pathways and molecules involved in DNA repair has been elusive but cannot be excluded.

The most important DNA lesions occurring after exposure of cells to ionizing radiation that determines death or survival of a cancer cell are the double strand breaks. A process called non-homologous end joining (NHEJ) repairs these lesions in eukaryote cells. Concerted concession of NHEJ requires the activity of the catalytic subunit of the DNA-dependent protein kinase (DNA-PKcs), which is a known substrate of caspase-3. In this study we therefore focused on the possibility that proteasome inhibition by MG-132 activates caspase-3, as has been reported previously [24,30]. This could mediate apoptosis and cause degradation of DNA-PKcs, the catalytic subunit of DNA-PK [20], resulting in radiosensitization. Reduction of DNA-PK activity following inactivation or mutation of DNA-PKcs is known to enhance radiosensitivity by decreasing repair of DNA-DSB's [59-61]. Although DSB repair is critical for cell survival after exposure to ionizing radiation [62], it is not clear whether this is a rate-limiting step dependent on the level of expression of DNA-PKcs. For example, DNA-PKcs level has been reported not to correlate with radiosensitivity of gliomas [63] and normal fibroblasts [64]. In any event, we were not able to detect meaningful changes in DNA-PK activity following MG-132 drug treatment. Degradation that did occur, appeared late at 24 hours and was probably an effect rather than a cause of the apoptotic process. According to the kinetics of the DSB-repair process described previously [65], any event interfering with the repair of DNA-DSB's has to take place during the initial 6 hours after irradiation. Consistent with the late cleavage of DNA-PKcs, we were not able to detect early activation of caspase-3 following treatment of PC-3 cells with MG-132. In fact, we observed a substantial drop in DEVD-AMC cleavage activity, which might be explained by the observation, that proteasome activity is necessary to activate caspase-3 at least in some cells [66,67]. Our observations are in accordance with data from Hideshima and coworkers who also could not detect changes in DNA-PKcs levels or caspase-3 activation during the initial 6 hours of Valcade-treatment in multiple myeloma cells [68]. Additionally, Wu and coworkers excluded the involvement of caspases in apoptosis of MO7e cells following proteasome inhibition, as caspase inhibitors failed to prevent DNA fragmentation [20] and it is therefore possible that proteasome inhibition induces caspase-independent apoptosis as described for cells, defective in the ubiquitin pathway [69].

Taken together, we conclude that although proteasome inhibition induces apoptosis in most cancer cells, sensitization of PC-3 cells to ionizing radiation occurs through mechanism that does not involve cleavage of DNA-PKcs.

## Competing interests

The author(s) declare that they have no competing interests.

## Authors' contributions

FP carried out the molecular studies and drafted the manuscript. AO and ChW participated in the design of the study and helped to draft the manuscript. WMB conceived of the study, and participated in its design and coordination and helped to draft the manuscript. All authors read and approved the final manuscript.

## Pre-publication history

The pre-publication history for this paper can be accessed here:
